# A Quality Improvement Project to Decrease Suboptimal Patient Transfers between Two Neonatal Units

**DOI:** 10.1097/pq9.0000000000000635

**Published:** 2023-02-13

**Authors:** Kiane A. Douglas, Chinonye Eriobu, Ann Sanderson, Dmitry Tumin, Uduak S. Akpan

**Affiliations:** From the * Department of Pediatrics, Brody School of Medicine, East Carolina University, Greenville, N.C.; † Division of Neonatology, ECU Health Medical Center, Greenville, N.C.

## Abstract

Poorly executed transitions of care in health care systems are associated with safety concerns and patient dissatisfaction. We noticed several problems in the transfer process between our neonatal intensive care unit (NICU) and special care nursery (SCN) and designed this quality improvement project to address them. The aim was to decrease suboptimal transfers from the NICU to the SCN by 50% over 9 months. We defined suboptimal transfers as the discharge of a patient within 3 days of transfer from the NICU to the SCN or the return of a patient to the NICU within 5 days of the transfer.

**Methods.:**

We formed a multidisciplinary team and collected baseline data from October 2019 to December 2020. Major interventions included implementing a transfer checklist and algorithm. We utilized 3 staff surveys to evaluate the progress of the project. We used statistical process control charts to track project measures over time.

**Results.:**

Patient demographics and SCN length of stay were similar for the baseline and postintervention periods. We decreased suboptimal transfers over 21 months (January 2021 to September 2022), achieved a significantly increased rate of parent notification before transfers (81% baseline versus 93% postintervention), and increased staff satisfaction with the transfer process (15% baseline versus 43% postintervention).

**Conclusions.:**

We successfully improved the transfer process from our NICU to the SCN via a quality improvement project. Increased staff satisfaction and the lack of perception of additional burden to the staff from the new process are expected to sustain our results.

## INTRODUCTION

Transitions of care are a critical part of patient care but could result in unintended consequences for patients if poorly executed.^1^ Transitions are associated with an increased risk of adverse events, care gaps, and communication lapses.^2,3,4,5^ Additionally, transitions can result in increased stress for patients and families, who report being frequently inadequately prepared for a transition or excluded from the discussion and decision-making process.^6,7^ One of the most frequent and difficult patient-care transitions occurs between critical care units and less acute settings,^8,9^ particularly in the neonatal intensive care unit (NICU), where several transitions can occur within and between hospitals during a neonate’s hospital stay.^10^ However, we know little about optimizing the transfer process of infants in the NICU.

Our NICU medical team noticed several patient transition problems, mostly due to a lack of standardization. To address these problems, we designed a quality improvement (QI) project to improve transitions between our NICU and special care nursery (SCN) within the same hospital. By consensus, our NICU team defined suboptimal transfers as the discharge of a patient from the hospital within 3 days of transfer from the NICU or transfer back to the NICU within 5 days of the transfer. We considered discharge within 3 days suboptimal as the discharge process can often span several days, especially for patients who have been cared for in a critical care unit. Transitioning to a new unit for such a brief period could increase the risk of a poor-quality discharge, potentially increase postdischarge complications, and may not be necessary.^11,12^ We aimed to decrease the percentage of suboptimal transfers between the NICU and the SCN by 50% over 9 months and sustain this improvement over 6 months.

## METHODS

### Setting

The NICU at our institution is a level IV unit that provides critical care for about 1000 neonates with various pathologies yearly. We utilize an electronic medical record system (EMR; EPIC, Verona, Wis.) for our medical records. Frequently, neonates no longer require critical care in the NICU but are not ready for discharge as they still have needs that require hospitalization, such as thermoregulation via an incubator or feeding via nasogastric tube. As a result, we transfer such patients to our level II SCN or a local community hospital, depending on the neonate’s needs and family preferences. All infants admitted to the NICU are subsequently eligible for transfer to the SCN if they meet transfer criteria. We admit approximately 400 neonates to our SCN yearly. There are separate physician and nursing teams for the NICU and SCN. The medical team consists of a neonatology attending, a neonatology nurse practitioner, and occasionally a neonatology fellow. Transfers occur during both the day and nighttime. Following a transfer to the SCN, the SCN physician team makes the medical decisions for the patients, including decisions to discharge home or return to the NICU. The decision to transfer back to the NICU is influenced by SCN staff training and unit level of care limitations.

There are usually several candidates eligible for transfer to SCN. Thus, carefully selecting candidates is important to decrease turnover rates while maintaining appropriate nursing staffing levels. We noticed that some neonates were transferred to the SCN and discharged home the next day, or returned to the NICU within a few days for worsened clinical status. A structured transfer process was lacking, so it was not always clear how we determined suitable neonates for transfer, who selected a patient for transfer, and who was responsible for informing the parents. Occasionally, the transfer decision was made by health care staff, such as pediatric residents, who may not have been fully qualified to do so independently. We rarely completed transfer notes, and if we informed families about transfers, the bedside nurse was the most likely to have done so. Before this project, the nurses documented parental notification in a flow sheet in the EMR. Nursing handoff occurred consistently, but physician handoff occurred only in the case of a complex patient.

### Interventions

**Fig. 1.**
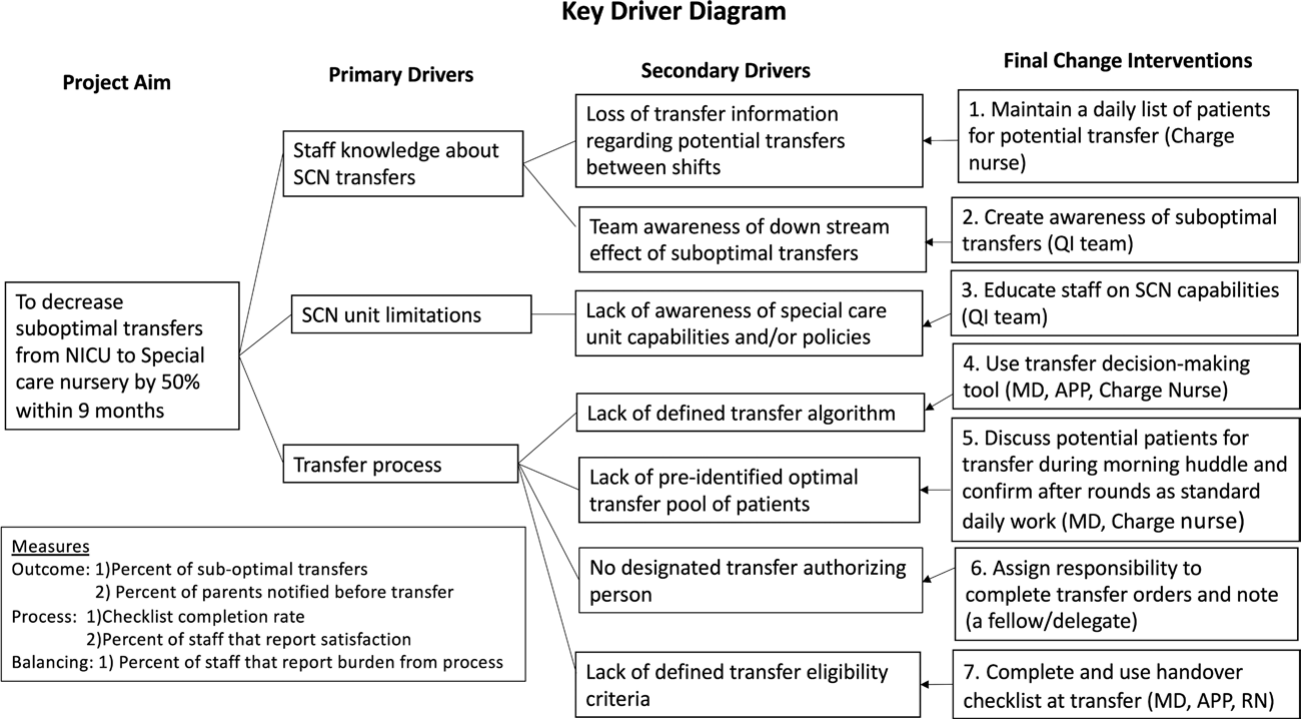
KDD.

We formed a multidisciplinary team consisting of a neonatologist, a neonatology fellow, a SCN neonatal advanced practice provider (APP), NICU and SCN charge nurses, and a medical student to improve the transfer process between the NICU and SCN in our institution. During the baseline period, we completed a fishbone analysis of the transfer process and designed a key driver diagram (KDD; Fig. 1). In addition, we identified primary drivers, including staff knowledge about transfers to SCN and SCN unit limitations regarding patient clinical status. We collected baseline data from October 2019 to December 2020. The intervention phase started in January 2021. The QI team introduced the project to care team members during team meetings and via email and distributed a baseline survey to physicians, APPs, and charge nurses in the NICU and SCN. We then introduced a checklist that included eligibility criteria for transfers to SCN and initial steps to be completed by a physician or APP before transfer. (**See Supplemental Digital Content, Appendix 1,** which shows checklist depicting transfer eligibility criteria for optimal transfer, candidates and steps to be completed prior to patient transfer. http://links.lww.com/PQ9/A457.)

Based on the baseline survey results and feedback from the first few months of the project [plan-do-study-act (PDSA) cycle 1], we created an algorithm to outline a step-by-step transfer process. (**See Supplemental Digital Content, Appendix 2**, which shows algorithm mapping out transfer process, http://links.lww.com/PQ9/A458.) We introduced the algorithm in March 2021, at the start of PDSA cycle 2. A huddle occurs between the medical team and the charge nurse before the beginning of daily rounds. As part of the algorithm, we recommended creating a list of patients eligible for the SCN and ranking patients in order of appropriateness for transfer during this huddle. Both nursing and physician day teams passed this information on to night teams, so the teams had the same list of candidates for transfer. When a candidate was ready for transfer, the neonatology fellow would ensure that all checklist steps had been completed before transfer orders were placed. We distributed a repeat survey during the intervention phase in May 2021. We modified the checklist and algorithm for PDSA cycle three starting in July 2021. In addition to introducing the modified documents, we included the bedside nurses in completing and verifying the checklist and encouraged them to use it during handovers. We also posted the transfer algorithm in key areas in the unit, such as the residents’ work room, the APP work room, and the charge nurse’s station. Finally, we created a smart phrase (a shortcut that links to a note template) in the EMR to document transfers, allowing for uniform documentation. This template documented the reason for the transfer, whether the team completed a transfer checklist, and informed the family of the transfer. Parental documentation required by this QI project supplemented the existing nursing documentation in the EMR. We administered a final survey at the end of the intervention period in September 2021. We monitored the process and collected data until September 2022, with final change interventions shown in the KDD.

### Measures

#### Outcome Measures

The QI team tracked the monthly percentage of suboptimal transfers as our primary outcome. We tracked the monthly percentage of parents notified before transfer as the secondary outcome measure, with a 95% notification rate target.

#### Process Measures

We selected the monthly percentage of patients with a completed transfer checklist as our primary process measure and the percentage of staff who reported satisfaction with the transfer process (at each survey round) as the secondary process measure with a target of 30%. The QI team deemed the enthusiastic response “very satisfied” superior to the lukewarm response “satisfied” and more likely to support sustained results.

#### Balancing Measure

The team agreed on the percentage of staff reporting the transfer process as “not at all burdensome” as the balancing measure, with a goal of ≥50%.

#### Data Collection

The QI team collected baseline data retrospectively from the EMR over 15 months, from October 2019 to December 2020. We collected postintervention data soon after each transfer occurred, over 15 months, with the initial 9 months representing the intervention period and a further 6 months to monitor for change sustainment. As we noted a trend toward achieving our primary outcome at the end of 15 months, we collected primary outcome data for an additional 6 months. We used a data sheet designed for this project and subsequently transferred data to an Excel spreadsheet for analysis. We designed and distributed surveys via SurveyMonkey (Momentive Inc., San Mateo, Calif.) or the Research Electronic Data Capture tool (Vanderbilt University, Tenn.).^13^ The QI team decided upon the questions by consensus. (**See Supplemental Digital Content, Appendix 3,** which shows survey questions distributed to staff to evaluate the project progress, http://links.lww.com/PQ9/A459.) We left the survey open for 2 weeks and sent out reminders via email 1 week after opening the survey.

#### Analysis

We tracked project measures monthly using statistical process control charts, with special cause variation defined based on previously described rules.^14^ We summarized patient characteristics as counts with percentages or medians with interquartile ranges (IQRs) and compared baseline and postintervention periods using rank-sum or chi-square tests. Results from the second and third rounds of the survey were each compared with the baseline survey round using Chi-square tests or Fisher exact tests, as appropriate. We considered *P* < 0.05 to be statistically significant. We performed data analysis with Stata 16.1 (College Station, Tex.: StataCorp, LP) and Excel (Redmond, Wash.: Microsoft Corporation).

#### Ethics

The institutional review board at our institution evaluated this project and deemed it not human subjects research.

## RESULTS

**Fig. 2.**
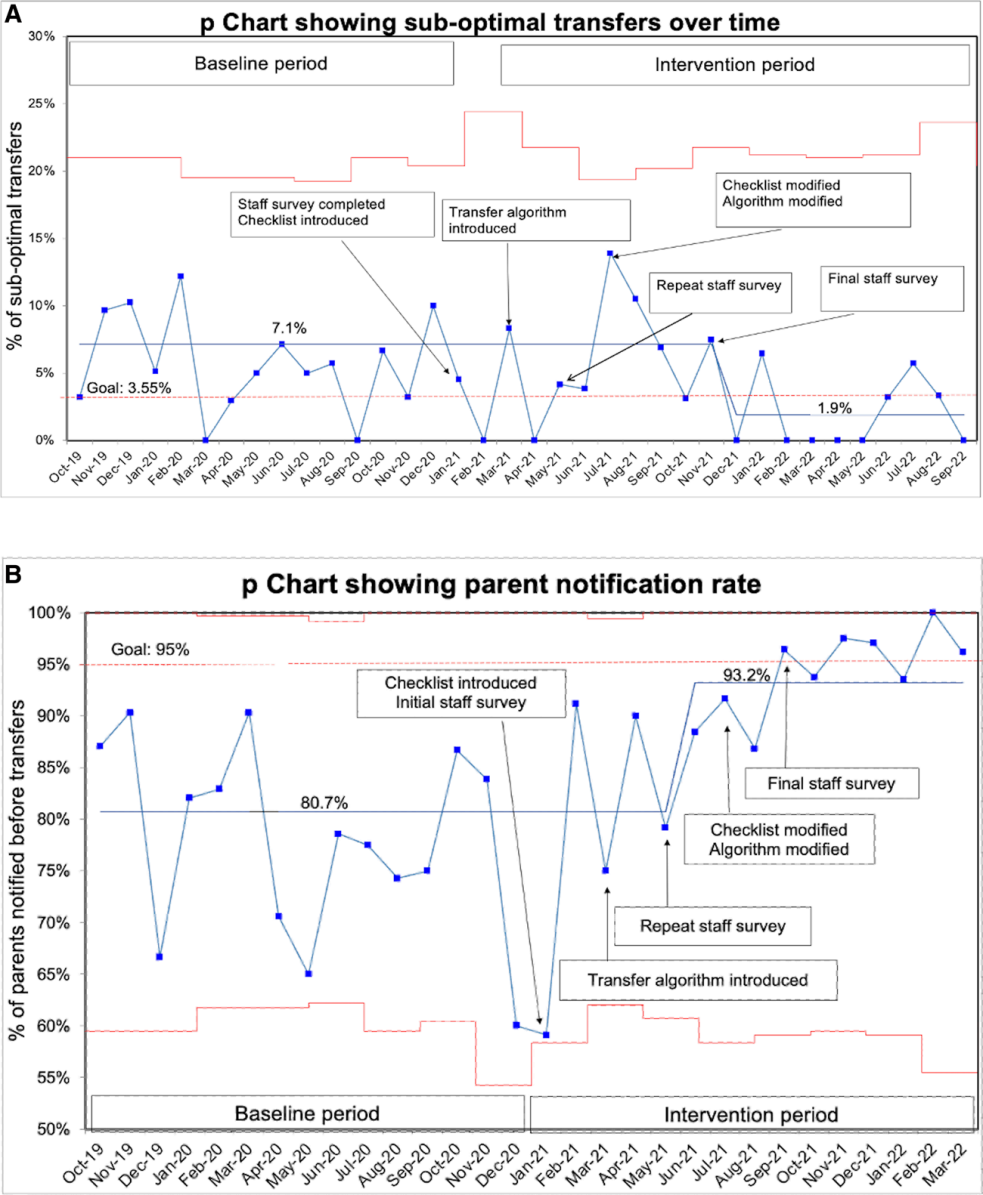
Outcome measures. A, p chart depicting the percentage of suboptimal transfers over the project duration. B, p chart depicting the percentage of parent notification before transfers over the project duration.

We collected data for 488 patients in the baseline period and 458 postintervention. The groups were similar regarding gestational age, birth weight, and length of stay in the SCN. We summarized the characteristics of the cohorts in Table 1. The principal reason for transfer to the SCN was the inability to ingest full feeds orally (81%), with neonates frequently having more than one reason for transfer. The p-chart showed that the centerline for suboptimal transfers decreased from 7.1% in the baseline period to 1.9% after December 2021 (Fig. 2A). Quick discharges accounted for a greater percentage of the suboptimal transfers than back transfers, 68% versus 32%. (**See Supplemental Digital Content, Appendix 4,** which shows bar graph depicting reasons for suboptimal transfers, http://links.lww.com/PQ9/A460.) Although we did not collect quantitative data on reasons for back transfer to the NICU, the most common reason for this at our center is increased respiratory support.

**Table 1.** Patient Characteristics in Baseline and Intervention Periods

Variable	Baseline Period (N = 488)	Intervention Period (N = 458)	*P*
	N (%) or Median (IQR)	N (%) or Median (IQR)	
Gestational age (wk)	32 (30–34)	33 (30–34)	0.894
Birth weight (g)	1742 (1266–2221)	1750 (1270–2170)	0.868
SCN length of stay (d)	15 (8–26)	14 (8–26)	0.717
Reason for transfer*			
Supplemental heat	N/A	234 (51)	
Bradycardia monitoring	N/A	21 (5)	
Learning to feed by mouth	N/A	369 (81)	

*Data not collected in baseline period.

N/A, not available.

Survey response rates (out of 93 potential respondents) were 58% at baseline, 44% at intervention I, and 49% at intervention II. The baseline survey revealed that there was neither a clearly defined transfer process nor a designated person responsible for transfer decisions. Furthermore, the team did not consistently notify families before transferring patients out of the NICU. The intervention I survey showed a persistent lack of awareness of the transfer process and the need to modify the eligibility criteria and the algorithm. The intervention II survey results indicated subsequent improvement in the transfer process (Table 2), with significantly more staff reporting the presence of a clearly defined process (*P* = 0.02). We collected data for 21 months following project initiation, achieving our primary goal in December 2021 (Fig. 2A). We also found significant improvement in the proportion of parents notified before the transfer, with a shift in the centerline of the chart showing parent notification rates (Fig. 2B).

**Table 2.** Survey Responses by Time Point

Survey Question/Response	Baseline (N = 54)	Intervention I (N = 41)	Intervention II (N = 46)	*P*	
	N (%)	N (%)	N (%)	Intervention I versus Baseline	Intervention II versus Baseline
Agree that the NICU has a clearly defined process for transfer to SCN*	25 (46)	24 (60)	35 (76)	0.421	0.002
Agree that there is a clear person responsible for transfer decisions	24 (44)	22 (54)	29 (63)	0.373	0.063
Agree that parents are always notified before SCN transfer*	13 (24)	23 (58)	33 (72)	0.001	<0.001
Is the transfer process burdensome?				0.287	0.550
Not at all	27 (50)	27 (66)	28 (61)		
Somewhat	23 (43)	13 (32)	16 (35)		
Very	4 (7)	1 (2)	2 (4)		
Satisfaction with the transfer process				0.204	0.003
Not at all	3 (6)	3 (7)	1 (2)		
Somewhat satisfied	43 (80)	26 (63)	25 (54)		
Very satisfied	8 (15)	12 (29)	20 (43)		

*Data missing for 1 case in intervention I survey.

**Fig. 3.**
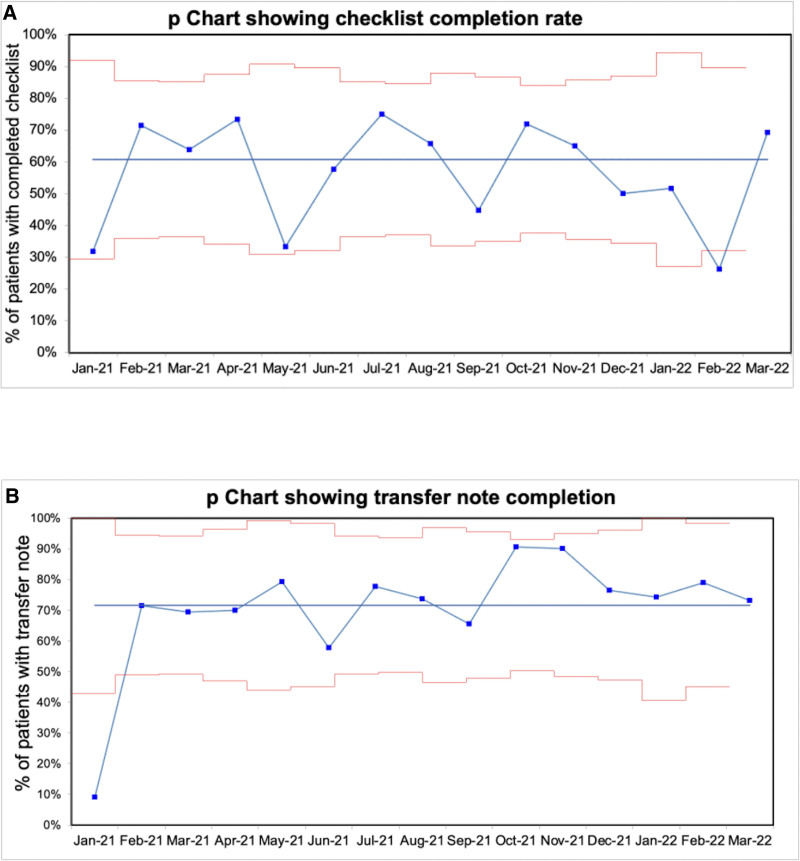
Process measures. A, p chart depicting the percentage of checklist completion during the project intervention phase. B, This p chart shows the percentage of transfer note completion during the project intervention phase.

Checklist use was inconsistent throughout the project. During the first month, the team completed the checklist 30% of the time, increasing to a mean of 60% at the end of the project (Fig. 3A). We observed more consistency in the documentation of transfers in the medical record using the smart phrase. During the first month, we completed transfer notes only 10% of the time. This percentage increased to a mean of 70% and was consistently above 70% for the last 6 months of the project (Fig. 3B). At baseline, only 15% of staff reported being very satisfied with the transfer process. A final survey showed significantly more staff reporting being very satisfied, exceeding our goal of 30%. We also achieved our goal for the balancing measure, with 61% reporting no burden at the project end versus 50% at baseline.

## DISCUSSION

We achieved our goal of decreasing suboptimal transfers from the NICU to the SCN in our institution. In addition, we improved the transfer process, with the medical team consistently notifying families before transferring patients out of the NICU. As a result, a significant percentage of staff reported being very satisfied with the transfer process without perceiving the increased workflow burden associated with transfers.

Transitions of care are fraught with imperfections and contribute to anxiety in patients and families. Major components of ideal transitions include teamwork and standardization, and standardized transition processes increase the quality of care.^8,10,15^ Other centers have demonstrated that using a standardized decision tool can improve decision-making around transfers, with benefits including team communication and decreased readmission rates.^16^ Similarly, our checklist and algorithm helped select appropriate candidates and clearly outlined the steps before a transfer to the SCN. In critical care units, a high census can influence transfer decisions.^15,17^ We noticed that under such circumstances in our NICU, the medical team was more likely to make a snap decision about a transfer, leading to suboptimal patient selection, failure to inform parents before the transfer, and inadequate communication between teams. Creating a transfer list during the morning huddle and assigning the responsibility for transfers to the neonatology fellow eased the pressure surrounding particularly difficult transitions. As a result of these changes, we achieved a smoother transition process and a reduction in suboptimal transfers compared to the baseline.

An important barrier to effective transitions of care is poor communication and inadequate transfer of information between health care teams, leading to discontinuity of care.^1,2,12^ In such situations, a lack of ready access to adequate documentation of a patient’s condition could lead to confusion and safety concerns.^1,6^ In our NICU, communication during transitions was frequently verbal, with no documentation in the medical record. Therefore, we implemented a smart phrase in the medical record to document transfers. This note communicated the reason for the transfer and the steps completed as part of the transition process, including family notification. Family integration is crucial during care transitions, yet families frequently report feeling excluded and not being informed about impending transfers.^6,10,15,18^ Family involvement is especially important in pediatrics, where patients may be unable to advocate for themselves. In other studies, parents reported higher satisfaction with transfers when given the rationale for and ample notice before the transfer.^19^ Although we did not collect data on parental satisfaction, our project significantly increased how often we contacted families before transfers. Occasionally, we could not contact a family despite several attempts. In such cases, the transfer note alerted the SCN team that they now had the responsibility to ensure family notification.

## CHALLENGES/LIMITATIONS

Our team experienced a few challenges while implementing this project. (**Supplemental Digital Content, Appendix 5,** which shows challenges faced during project implementation, http://links.lww.com/PQ9/A461.) First, we conducted this project during the COVID-19 pandemic, with its attendant staffing and census challenges. There were quarantine requirements for staff and patients following exposure to the virus, resulting in sudden and unexpected staff shortages. It was often necessary to reassign patients according to nursing availability, including transferring patients to the SCN. At those times, the transfer process was sidelined, compromising the primary project outcome. Second, we noted inconsistent use of the transfer checklist throughout the project. The checklist is a paper document, and there were difficulties with its availability and easy access. Discussions with health care providers indicated that a checklist incorporated into the EMR would be easier to use, and we plan to implement this change. Finally, our neonatal units are proximally located within the same building, potentially leading to less strict adherence to transfer steps than if the units were located in different facilities. This fact may explain our project’s 32% back-transfer rate compared to a 20% readmission rate reported elsewhere.^20^ The main strength of this project is that we achieved a patient-centered approach to transfers of care that can easily be replicated by other NICUs and customized to suit their needs. In addition, we paid attention to staff satisfaction to ensure decreased staff burnout, project sustainability, and patient safety eventually. We also acknowledge some limitations, including a lack of parental satisfaction data and data on hospital readmission after SCN discharge. Increased readmission rates have been associated with transfers to step-down units before discharge, although failed transfers in these analyses also included back transfers to the NICU.^21,22^

## CONCLUSIONS

Transitions of care can be especially difficult from a critical care unit, such as a NICU, to a less acute setting. Poorly executed transitions have serious implications for patient care, and ensuring effective transitions is crucial for patient safety. Including all unit stakeholders during the planning phases of a transition process can achieve a standardized, satisfactory system for staff and patients without perceived unnecessary burden.

## ACKNOWLEDGMENT

We thank Kelly Allis, Kathy Barnhill, and Elaine Henry for contributing to this project.

## DISCLOSURE

The authors have no financial interest to declare in relation to the content of this article.

## Supplementary Material
