# Supplementary material for: A Quality Improvement Project to Decrease Suboptimal Patient Transfers between Two Neonatal Units
Source: Pediatr Qual Saf. 2023 Feb 13;8(1):e635. doi: 10.1097/pq9.0000000000000635 (PMC9925099; doi:10.1097/pq9.0000000000000635)
Supplement: Supplementary file 1 [file pqs-8-e635-s001.pdf]

A Quality Improvement Project to Decrease Suboptimal Neonatal Transfers from an Intensive Care Unit to a Special Care Nursery
First author: Kiane A. Douglas, BS

Supplemental Digital Content, Appendix 1. Transfer checklist

- Please ensure checklist is complete upon transfer
- All team members may contribute to completion of checklist

Optimal Candidate for SCN		Checklist PRIOR to transfer	
	Respiratory support < 4 lpm		Team rounded on patient with plan to transfer to SCN
	Brady count down		Daily physical assessment completed
	Need to work on PO feeding		Transfer orders entered / reconciled old orders
	Need to work on thermoregulation		Discussion with primary RN / charge RN
	Need for continued therapy (ie: antibiotic completion, opioid wean)		Parent notification
	Not requiring frequent, invasive procedures		Transfer note in chart
	Not a candidate for transfer closer to home		Fellow MD on-call aware of transfer

FIGURE LEGENDS

Figure 1. Key Driver Diagram

Figure 2. Outcome Measures

Figure 3. Process Measures

SUPPLEMENTAL DIGITAL CONTENT

Supplemental Digital Content, Appendix 1. Transfer Checklist

Supplemental Digital Content, Appendix 2. Transfer Algorithm

Supplemental Digital Content, Appendix 3. Survey Questions

Supplemental Digital Content, Appendix 4. Breakdown of Suboptimal Transfers

Supplemental Digital Content, Appendix 5. Barriers to Project Implementation
