# Supplementary material for: A Quality Improvement Project to Decrease Suboptimal Patient Transfers between Two Neonatal Units
Source: Pediatr Qual Saf. 2023 Feb 13;8(1):e635. doi: 10.1097/pq9.0000000000000635 (PMC9925099; doi:10.1097/pq9.0000000000000635)
Supplement: Supplementary file 2 [file pqs-8-e635-s002.pdf]

A Quality Improvement Project to Decrease Suboptimal Neonatal Transfers from an Intensive Care Unit to a Special Care Nursery
First author: Kiane A. Douglas, BS

Supplemental Digital Content, Appendix 2. Transfer algorithm

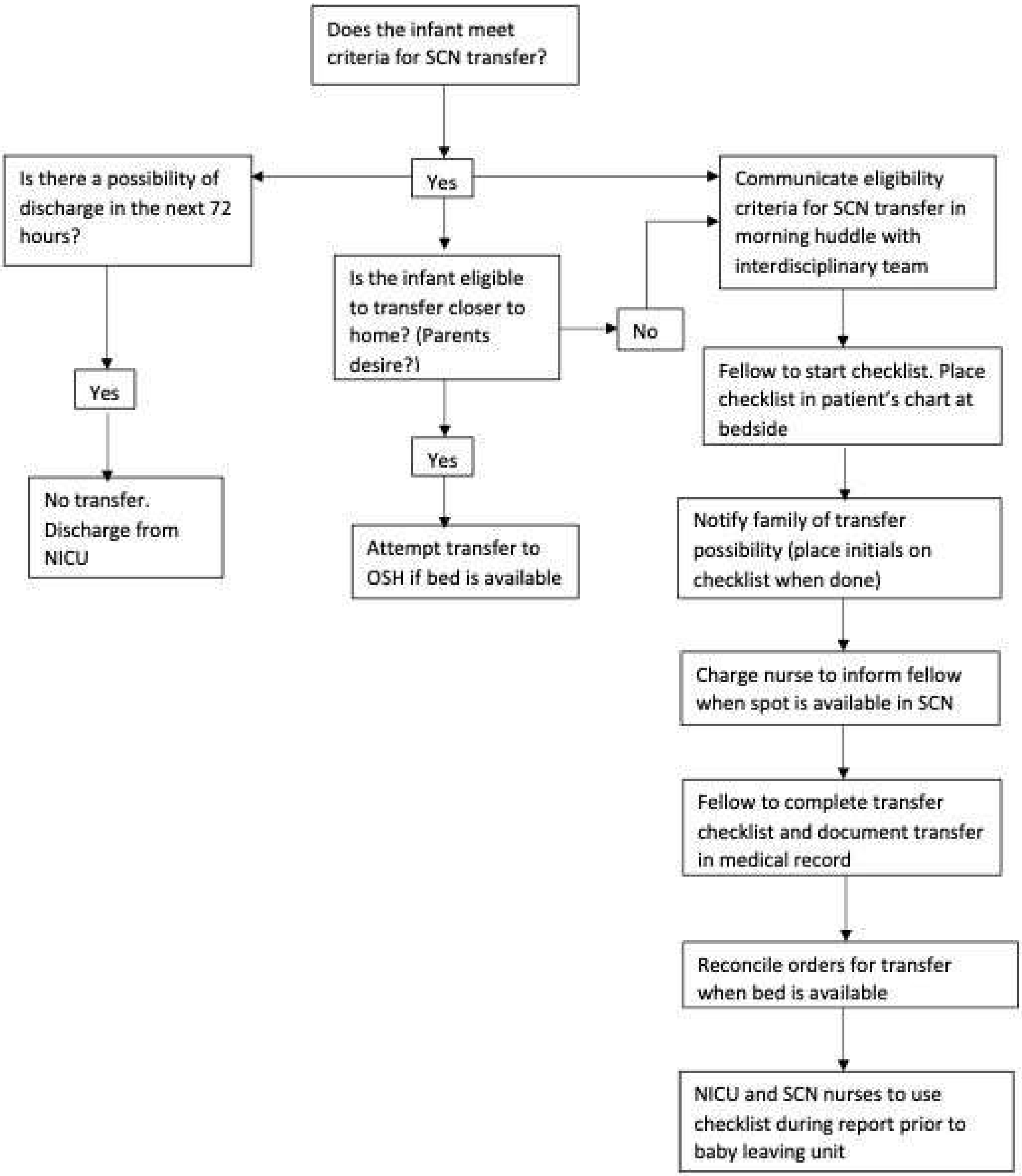

FIGURE LEGENDS

Figure 1. Key Driver Diagram

Figure 2. Outcome Measures

Figure 3. Process Measures

SUPPLEMENTAL DIGITAL CONTENT

Supplemental Digital Content, Appendix 1. Transfer Checklist

Supplemental Digital Content, Appendix 2. Transfer Algorithm

Supplemental Digital Content, Appendix 3. Survey Questions

Supplemental Digital Content, Appendix 4. Breakdown of Suboptimal Transfers

Supplemental Digital Content, Appendix 5. Barriers to Project Implementation
