# Supplementary material for: A Quality Improvement Project to Decrease Suboptimal Patient Transfers between Two Neonatal Units
Source: Pediatr Qual Saf. 2023 Feb 13;8(1):e635. doi: 10.1097/pq9.0000000000000635 (PMC9925099; doi:10.1097/pq9.0000000000000635)
Supplement: Supplementary file 3 [file pqs-8-e635-s003.pdf]

NICU-SCN Transfer Survey

1) Does the NICU have a clearly defined process for transferring patients from the NICU to SCN? Y/N

2) Are you familiar with the eligibility criteria for intermediate care (SCN) patients? Y/N

3) Do you think there is a clear selection process for potential SCN candidates? Y/N

4) How are patients selected for transfers?
   a) From list generated at huddle
   b) Charge nurse preference
   c) Physician preference
   d) Not sure

5) Is there a clear person responsible for transfer decisions? Y/N

6) Parents are always notified of transfer to SCN prior to transfer Y/N

7) Do you consider the transfer process to be burdensome?
   a) Not at all
   b) Very
   c) Somewhat

8) How satisfied are you with the transfer process between NICU and SCN?
   a) Not at all
   b) Somewhat satisfied
   c) Very satisfied

9) Discipline of respondent
   - Physician
   - APP
   - NICU charge
   - SCN charge

FIGURE LEGENDS

Figure 1. Key Driver Diagram

Figure 2. Outcome Measures

Figure 3. Process Measures

SUPPLEMENTAL DIGITAL CONTENT

Supplemental Digital Content, Appendix 1. Transfer Checklist

Supplemental Digital Content, Appendix 2. Transfer Algorithm

Supplemental Digital Content, Appendix 3. Survey Questions

Supplemental Digital Content, Appendix 4. Breakdown of Suboptimal Transfers

Supplemental Digital Content, Appendix 5. Barriers to Project Implementation
