# Supplementary figures and images for: A Quality Improvement Project to Decrease Suboptimal Patient Transfers between Two Neonatal Units
Source: Pediatr Qual Saf. 2023 Feb 13;8(1):e635. doi: 10.1097/pq9.0000000000000635 (PMC9925099; doi:10.1097/pq9.0000000000000635)

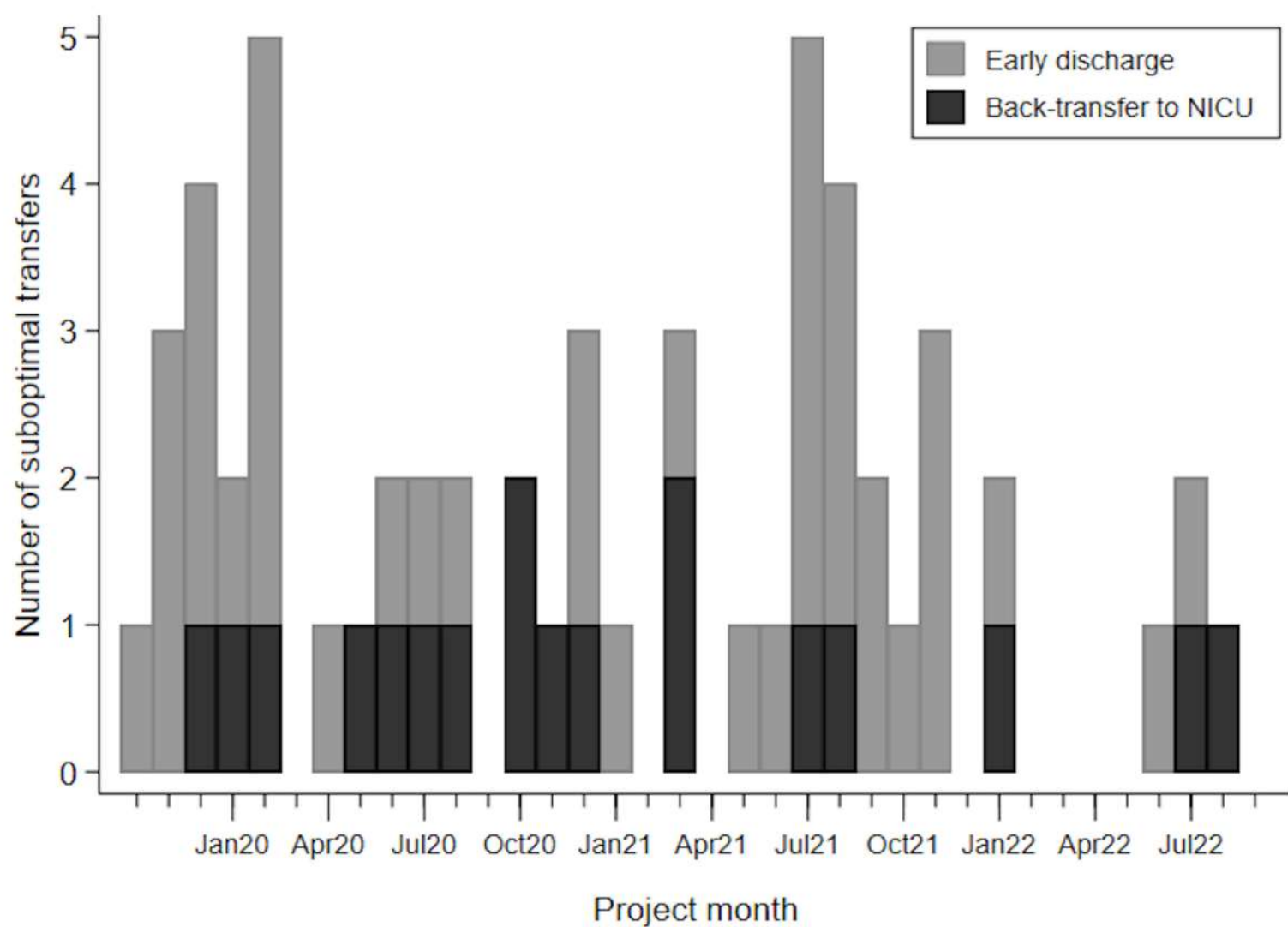

Supplement: Supplementary file 4 [file pqs-8-e635-s004.pdf]
