# Supplementary material for: A Quality Improvement Project to Decrease Suboptimal Patient Transfers between Two Neonatal Units
Source: Pediatr Qual Saf. 2023 Feb 13;8(1):e635. doi: 10.1097/pq9.0000000000000635 (PMC9925099; doi:10.1097/pq9.0000000000000635)
Supplement: Supplementary file 5 [file pqs-8-e635-s005.pdf]

**Table 3**: Barriers to Project Implementation

| **Barriers** | **Mitigation strategies** | **Lessons learned** |
|---|---|---|
| Paper checklist use | Temporary smartphrase in electronic medical record (EMR). | Easy accessibility and fewer steps encourage adoption of changes. In this case, completing the checklist in the EMR was more desirable than printing off a list and completing by hand. Building the checklist directly into the EMR would further encourage checklist completion. |
| Special Care Nursery located within the same hospital | Regular staff updates highlighting data on suboptimal transfers and emphasizing the downsides of suboptimal transfers. | As the SCN was located within the same hospital, back-transfers did not seem as consequential compared to readmission from a community hospital. Thus, the medical team did not put the same effort into optimizing transfers to SCN as was the case for transfers to community hospitals. |
| COVID-19 pandemic quarantine requirements and staffing challenges | The QI team encouraged the medical team to follow the transfer algorithm as much as possible despite the challenges. | Occasionally, extenuating factors place considerable strain on workflow. Well-established practices may be able to withstand the challenges, but new processes that are not yet part of the culture are at risk of being sidelined. |

## FIGURE LEGENDS

**Figure 1**. Key Driver Diagram

**Figure 2.** Outcome Measures

**Figure 3.** Process Measures

## SUPPLEMENTAL DIGITAL CONTENT

**Supplemental Digital Content, Appendix 1.** Transfer Checklist

**Supplemental Digital Content, Appendix 2.** Transfer Algorithm

**Supplemental Digital Content, Appendix 3.** Survey Questions

**Supplemental Digital Content, Appendix 4.** Breakdown of Suboptimal Transfers

**Supplemental Digital Content, Appendix 5.** Barriers to Project Implementation
